# Changes in on-time vaccination following the introduction of an electronic immunization registry, Tanzania 2016-2018: interrupted time-series analysis

**DOI:** 10.1186/s12913-022-08504-2

**Published:** 2022-09-20

**Authors:** Samantha B. Dolan, Roy Burstein, Jessica C. Shearer, Ngwegwe Bulula, Hil Lyons, Emily Carnahan, Emily Beylerian, Jenny Thompson, Nancy Puttkammer, William B. Lober, Shan Liu, Skye S. Gilbert, Laurie Werner, Tove K. Ryman

**Affiliations:** 1 Dolan Consulting LLC, PATH, Seattle, USA; 2 Department of Global Health, University of Washington, Seattle, USA; 3 Present Address: Bill and Melinda Gates Foundation, Seattle, 98109 USA; 4 Institute for Disease Modeling, Bellevue, USA; 5 PATH, Seattle, USA; 6 Immunisation and Vaccine Development Program, Ministry of Health, Community Development, Gender, Elderly and Children, Government of Tanzania, University of Dodoma, Dodoma, Tanzania; 7 Biobehavioral Nursing and Health Informatics, University of Washington, Seattle, WA USA; 8 Department of Industrial and Systems Engineering, University of Washington, Seattle, WA USA

**Keywords:** Electronic immunisation registry, Vaccination timeliness, Routine health information system, Digital health intervention, Interrupted time-series analysis

## Abstract

**Background:**

Digital health interventions (DHI) have the potential to improve the management and utilization of health information to optimize health care worker performance and provision of care. Despite the proliferation of DHI projects in low-and middle-income countries, few have been evaluated in an effort to understand their impact on health systems and health-related outcomes. Although more evidence is needed on their impact and effectiveness, the use of DHIs among immunization programs has become more widespread and shows promise for improving vaccination uptake and adherence to immunization schedules.

**Methods:**

Our aim was to assess the impact of an electronic immunization registry (EIR) using an interrupted time-series analysis to analyze the effect on proportion of on-time vaccinations following introduction of an EIR in Tanzania. We hypothesized that the introduction of the EIR would lead to statistically significant changes in vaccination timeliness at 3, 6, and > 6 months post-introduction.

**Results:**

For our primary analysis, we observed a decrease in the proportion of on-time vaccinations following EIR introduction. In contrast, our sensitivity analysis estimated improvements in timeliness among those children with complete vaccination records. However, we must emphasize caution interpreting these findings as they are likely affected by implementation challenges.

**Conclusions:**

This study highlights the complexities of using digitized individual-level routine health information system data for evaluation and research purposes. EIRs have the potential to improve vaccination timeliness, but analyses using EIR data can be complicated by data quality issues and inconsistent data entry leading to difficulties interpreting findings.

**Supplementary Information:**

The online version contains supplementary material available at 10.1186/s12913-022-08504-2.

## Introduction

As information technology becomes more accessible, health programs are adopting digital health interventions (DHIs) to improve the provision and demand for health services [1]. DHI have the potential to improve the management and utilization of health information to optimize health care worker (HCW) performance and provision of care, and ultimately improve health outcomes. The importance of digital technologies was highlighted in 2018 by the World Health Assembly, noting that these tools could be integrated into existing health systems and scaled-up to help achieve the Sustainable Development Goals [2]. Despite the proliferation of DHI projects in low-and middle-income countries (LMICs), few have been evaluated to understand their impact on health systems and health-related outcomes [1, 3, 4]. Although more evidence is needed on the impact and effectiveness of DHIs within existing healthcare settings, the use of DHIs among immunization programs has become more widespread and shown promise for improving vaccination uptake and adherence to immunization schedules [5–7].

Globally 86% of children in 2018 received recommended childhood vaccinations, falling below the Global Vaccines Action Plan national goal of 90% [8, 9]. In Africa, 84% of children received their first dose of pentavalent vaccine, while only 76% received the third dose in the recommended series [10]. Receiving a vaccination on-time ensures a child has an optimal immune response and that they can be protected from vaccine-preventable diseases as quickly as possible [11]. To better track children’s vaccination histories and identify those children behind on their schedules, electronic immunization registries (EIRs) have been introduced among some immunization programs in LMICs to replace health facilities’ paper-based tools. EIRs are “confidential, population-based and computerised systems that collect vaccination data about residents within a geographic area or with a healthcare provider” and allow for the monitoring of vaccination coverage by provider, vaccine, dose, age, target group, and geographical area, and facilitate the monitoring of individuals receiving immunizations [12–14].

DHIs have ushered in a data “revolution” that introduces new possibilities, and potential hurdles, for public health research [15]. They have the potential to improve data quality and provide greater insight into program performance as they aim to efficiently capture and report standardized, individual-level health data [6, 14, 16–19]. The global community has recommended that the effectiveness of EIRs for monitoring immunization programs be demonstrated in comparison to existing methods [20]. However, despite the potential of EIRs, the best approach to using data from these tools for analytics has yet to be determined. They offer a promising change from the typical use of routine surveys and/or ad-hoc efforts to collect information in these settings and instead allow health programs with few resources to leverage existing, routinely collected data [15]. We attempted to fill this gap in the utility of DHI by demonstrating how EIR data could be used to assess vaccination timeliness.

EIRs can hypothetically improve accessibility of vaccination data by HCWs, therefore these systems should allow HCWs to identify vaccines due more easily and follow-up with defaulters, thereby improving both immunization timeliness and coverage [21, 22]. One study based in Vietnam found that an EIR and a short-message service (SMS) reminder system improved coverage, however there are few studies that have assessed the effectiveness of EIRs on immunization timeliness [6]. For this study, our objective was to assess the impact of a DHI on immunization timeliness by using an interrupted time-series analysis to investigate the proportion of on-time vaccinations following introduction of an EIR in Tanzania. We were also interested in showcasing how individual-level routine health information system (RHIS) data from EIRs could be analyzed and used for future research purposes.

### Study context and intervention description

The Better Immunization Data (BID) Initiative partnered with the Ministry of Health (MOH) in Tanzania to address key challenges in immunization data collection, quality, and use beginning in 2013 [23]. The government identified areas of concern at the outset of the project were poor data quality, inaccurate denominator data, defaulter tracing, poor data visibility, complex data systems, and inadequate data management and use [24]. Digital technologies can help overcome these types of health system challenges, particular through interventions built for clients and health care providers [1].

**Fig. 1:**
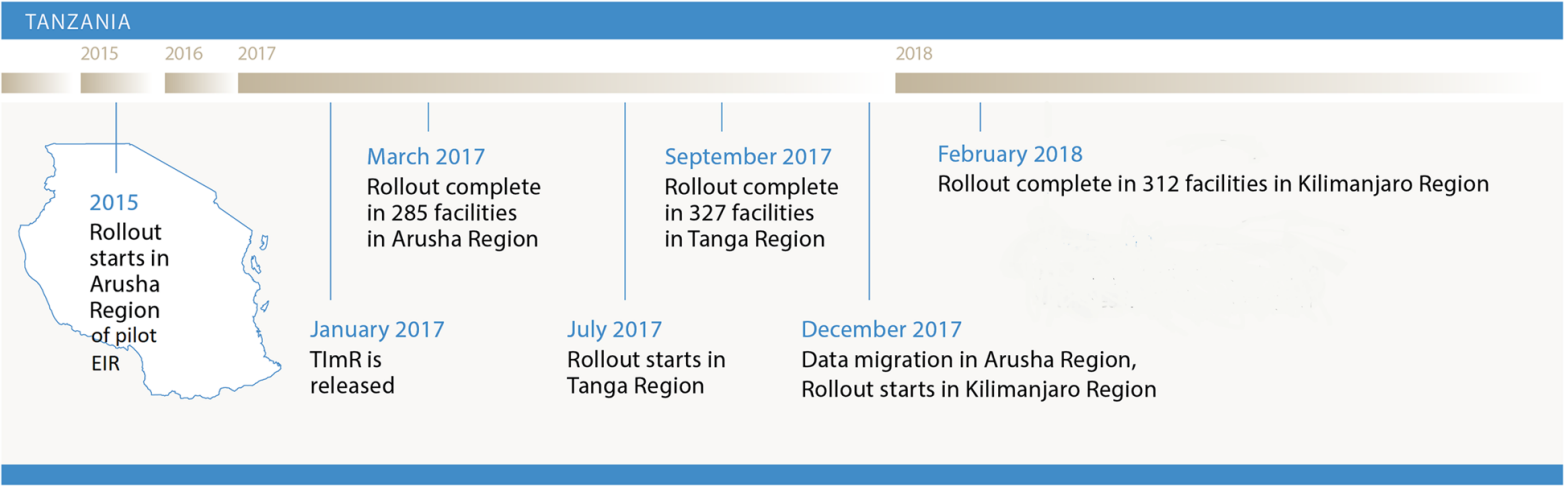
Timeline of EIR Introduction. Note: TImR: Tanzania Immunization Registry

The BID Initiative implemented an intervention package to address the challenges identified, including: establishment of user-advisory groups (UAGs); development of tablet-based EIR software, the establishment of the Tanzania Immunization Registry (TImR), with online and offline functionality that enabled automated, simplified reports; development of logistics management information systems; provision of targeted supportive supervision for HCWs; establishment of peer support networks (via WhatsApp groups); and support for a data-use culture [24]. By 2018, the package was deployed in the regions of Arusha, Dodoma, Kilimanjaro, and Tanga of Tanzania (Fig. 1). Project staff used a phased roll-out approach to introduce the intervention package to each district within these regions, starting with Arusha as the pilot site which implemented a different EIR before migrating to TImR. Completing paper-based forms and reports remained a requirement by the MOH throughout the project; therefore, all facilities completed dual data entry from the time of EIR introduction and onwards. The EIR allowed HCWs to register children, record vaccinations administered, quickly identify vaccinations due, and generate aggregate facility-level reports that fed into the health management information system. The intention of the EIR was to replicate and eventually replace the use of paper-based data collection tools used in immunization clinics, and in March 2018 facilities in the Tanga region of Tanzania started transitioning to entirely digital reporting as the MOH in this region was comfortable removing the paper-based reporting requirement. Additionally, beginning in April 2018 SMS vaccination reminders were sent automatically through the EIR’s server to the caregivers of children with delayed vaccination visits.

Upon introduction of the EIRs, facilities underwent a data back-entry process where all records included in the facility’s paper-based vaccination register were input into the EIR in batches. HCWs were instructed to register each child seen for immunization services and to enter all vaccines the child had previously received, if they were not already recorded, to maintain a complete vaccination record for every child in the EIR. When children were seen for services at the facility, they were assigned unique patient identifiers which were printed on barcode labels and provided to caregivers, so records could be quickly retrieved upon a child’s subsequent visit at any facility using the EIR. Vaccination information was entered into the EIR for registered children returning to the facility either at the point-of-care (POC) or retrospectively on the same day after the immunization clinic ended. Within the EIR records there is no indication of whether a record was back entered.

### Theoretical background

**Fig. 2:**
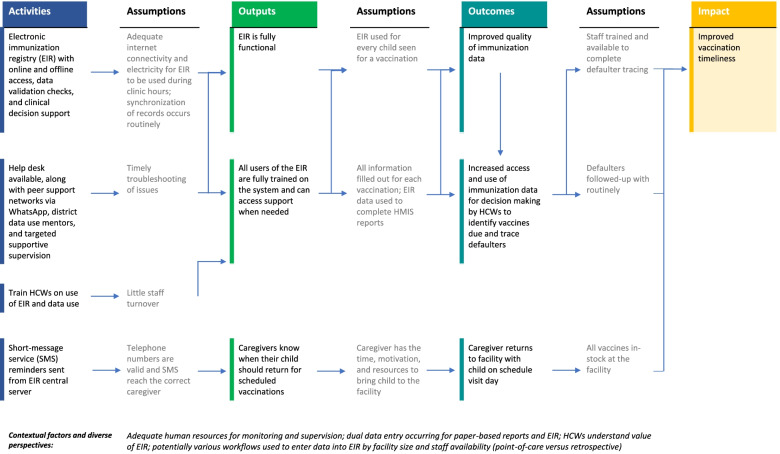
Casual linkage diagram of the relationships and assumptions between implementation of an electronic immunization registry and improved vaccination timeliness

Our causal linkage diagram is specific to our study, including key assumptions, and describes our theory of change for the EIR (Fig. 2), however a separate theory was developed for the BID intervention package [25]. Briefly, we believed that by introducing the BID intervention package, HCWs should have improved access to and use of data to identify vaccines due and follow-up with defaulters, therefore vaccination timeliness should improve. This theory makes the following assumptions: a) every child seen at a facility is entered into the EIR; b) all required information is entered into the system; and c) HCWs actively complete follow-up with defaulters, including the use of automated SMS-reminders.

## Methods

### Study design

We used secondary data from the EIR as implemented in over 1000 health facilities to test the following hypothesis: the introduction of the EIR in Tanzania lead to improvement in vaccination timeliness after 3,6, and > 6 months post-introduction. We used an interrupted time-series (ITS) analysis study design to measure changes over time. We chose this design for several reasons. First, we could not use any experimental designs that randomized intervention sites on practical grounds, as the MOH and BID Initiative dictated where and when the intervention would be introduced, and our analysis began after implementation. Second, we had limited resources to collect new data, so we had to leverage existing routine health information system data and the EIR data. These constraints made a quasi-experimental study design like ITS a good choice as it is logistically easier to conduct than a randomized trial and minimizes threats to ecological validity [26].

### Primary analysis

We set up our primary analysis based on our hypothesized theory of change, but quickly found substantial levels of missingness in the data. Missingness could be either due to a child not receiving a vaccination or lack of recording. As such, we conducted a sensitivity analysis to attempt to address this limitation using only registered children with a subsequent vaccination dose recorded in the EIR as this likely meant their record had less missing information.

### Data acquisition and population of interest

All data available from the time the EIR was first used at each facility were pulled from the central EIR repository. Duplicate records, records with erroneous dates for vaccination or date of birth such as those with implausible or invalid dates, and records of children born before 2010 were all excluded. Only data from the regions of Arusha, Kilimanjaro, and Tanga were included since these regions had more than 6 months of experience with the EIR at the time of analysis; these data included EIR records from January 2015-September 2018. Facilities with fewer than 6 months of data were excluded because they did not have enough data to be assessed at our 6-month endpoint, also we assumed that these facilities were still adjusting to the EIR and not consistently using the system to capture vaccinations. Observations were excluded if the date of vaccination (real or planned) occurred after the data pull. Children > 2 years at the time of the data pull were excluded based on the assumption that their records may have been incomplete, since they were unlikely to visit a facility to receive vaccinations following introduction of the EIR. To assess time trends relative to EIR introduction across all implementation waves, time was centered relative to the date of EIR introduction for each health facility despite receiving the EIR at different times. Vaccinations administered before EIR introduction were captured from the retrospectively entered data; the original data were recorded in vaccination registers held at the facilities. Because we could not disentangle back-entered data from records entered in real-time, we relied on EIR-introduction date as a proxy.

### Outcome measure

We measured proportion of on-time vaccinations per facility per month for the 1st, 2nd, and 3rd doses of pentavalent vaccine, which covers diphtheria, tetanus, and whole-cell pertussis, hepatitis B, and *Haemophilus influenzae* type b (DTP); and 1st dose of measles-containing vaccine (MCV1). Timeliness of vaccination was based on each child’s date of birth for the first dose in a series or the date of the previous dose administered in a vaccination series, e.g., time between DTP2 and DTP3 vaccination was calculated to determine timeliness, while time since birth was used to determine timeliness of DTP1 and MCV1. We used the MOH’s programmatic definition of timeliness, vaccinations were considered on-time if they were administered within 7 days on the due date; doses administered early were included in our analysis, but not considered on-time.

### Statistical analysis

We summarized health facility characteristics by the number of children registered in the EIR, time since EIR introduction, primary power source, facility ownership (private or public), and facility type using data from RHIS, including the EIR. Counts and proportions were used to summarize facility characteristics within each region. Facility types were categorized based on government definitions; dispensaries were the lowest level of health service provision, health centers provide a wider range of services, and district or regional hospitals offer inpatient, outpatient, and specialized services [27]. Vaccination characteristics were summarized by vaccine type, region, and pre- or post-EIR time period. We calculated the number of eligible children per vaccine type based on a child’s age at the time of the EIR data pull in September 2018 and recommended vaccination schedule for Tanzania [28]. On-time vaccinations were summarized by individual-level data transformed into counts and proportions with the denominator as all eligible children. The number of days after the scheduled date among eligible children and number of vaccinations administered per facility per month were summarized using means and standard deviations. Vaccination history missingness was summarized by the number of children with no vaccinations recorded, no vaccinations beyond birth doses, those with a second or third dose recorded, but missing an earlier dose in a vaccine series, and those receiving one, but not all vaccines due at a single visit.

To estimate the changes in vaccination timeliness, we fit a hierarchical binomial model with a logit link at the level of the health facility, where the outcome was proportion of on-time visits per facility per month. Recall that we hypothesized that the introduction of the EIR would lead to changes in vaccination timeliness over time. We chose to assess the time periods of 0-3, 4-6, and > 6 months post EIR-introduction based on programmatic expectations for when EIR-users would become comfortable using a new technology at the POC and when we expected to see routine use of the system. To test this hypothesis we structured our model to estimate the immediate impact on the level of vaccination timeliness at each time period post EIR-introduction compared to pre EIR (level change) coded as a categorical variable, the slope of the change in successive months for each time period (slope change) was coded as sequentially numbered months during the time period and 0 before or after, and the secular trend in timeliness (time) was centered on the date of EIR-introduction as time 0 and coded sequentially throughout the entire study period. Random intercepts were included at the district and regional levels to account for clustering of observations.

The *p*_*ft*_ term represents our outcome of interest denoting the proportion of on-time vaccinations for each facility *f* (running from 1 to 937 facilities) at month *t* (running from − 16 to 27, with month centered on the date of EIR introduction); *β*_0_ estimates the mean level of the outcome; *β*_1 − 4_ are variables measuring slope changes for each time period *t* and facility *f*, *β*_1_(*Time*) estimates the baseline monthly secular trend in on-time vaccinations before EIR-introduction, *β*_2_ (*TimeAfterIntro3mos*) estimates the slope change during months 0-3 post EIR-introduction, *β*_3_ (*TimeAfterIntro6mos*) estimates the slope change for months 4-6 following EIR-introduction, and *β*_4_ (*TimeAfterIntrogt6mos*) estimates the slope change for > 6 months post-introduction; *β*_5 − 7_ are intercept variables measuring immediate level changes in the proportion of on-time vaccinations post EIR-introduction for each time period *t* and each facility *f* compared to time pre EIR*,* segmenting time for 0-3 months (*3mosAfterIntro*), 4-6 months (*6mosAfterIntro*), and > 6 months (*gt6mosAfterIntro*) following EIR-introduction. Random effects separately estimated the outcome for each district and region and were assumed to be normally distributed on the log-odds scale. No other covariates were included in the model for simplicity of interpretation.

$${\displaystyle \begin{array}{c}{Y}_{ft}\sim Binomial\left({p}_{ft},{N}_{ft}\right)\\ {} logit\left({p}_{ft}\right)={\beta}_0+{\beta}_1 Time_{ft}+{\beta}_2 TimeAfterIntro3 mos_{ft}+{\beta}_3 TimeAfterIntro6 mos_{ft}+{\beta}_4 TimeAfterIntrogt6 mos_{ft}+{\beta}_53 mosAfterIntro_{ft}+{\beta}_66 mosAfterIntro_{ft}+{\beta}_7 gt6 mosAfterIntro_{ft}+ u_{fR}+ u_{fD}\\ {}\begin{array}{c} u_{fR}\sim N\left(0,{\sigma}_R^2\right)\\ {} u_{fD}\sim N\left(0,{\sigma}_D^2\right)\end{array}\end{array}}$$

- *Y* − *number of on* − *time vaccinations*
- *N* − *elgible children*
- *p* − *proportion on time*
- *β* − *parameters for fixed effects*
- *u* − *normal independent and identically distributed random effects*
- *t – time (month for β*_1 − 4_ *and time period for β*_5 − 7_*)*
- *f – facility*
- *R- region*
- *D- district*

Due to the amount of missing data found during our primary analysis, we conducted a sensitivity analysis to understand if timeliness differed among children with multiple vaccinations recorded. To conduct this analysis, we estimated timeliness of DTP1 among children with a documented dose of DTP2 in the EIR, which could have been retrospectively or prospectively entered. For the sensitivity analysis, we used the same model, but estimated the proportion of on-time visits only for DTP1 per facility among only children that had received DTP2.

All analyses were conducted in RStudio (version 1.1). The glmer function in the R lme4 package was used to model our binomial outcome for the four vaccines of interest, weighted by the number of eligible children. Significance was determined at a two-sided alpha value of 0.05.

### Ethics

This study was determined to be non-human subjects research by PATH, as it is part of routine program evaluation. SD, RB, HL, and JS led the design, implementation, and interpretation of findings for this study and were not involved in the BID Initiative design or implementation.

## Results

### Facility characteristics

In Tanzania, 1006 facilities within 20 districts and 3 regions had initiated using the EIR and were included in this study, totaling 251,815 children with vaccinations recorded in the EIR (Table 1). At the time of data extraction from the EIR database, facilities had used the EIR from 9 to 27 months. The most used power source amongst facilities was grid power (37%). Most facilities were public, government-owned (75%) and were dispensaries (82%).

**Table 1:** Characteristics of health facilities using the electronic immunization registry (EIR) in Tanzania, by region^a^

Characteristics	Arusha	Kilimanjaro	Tanga	All Regions
Number of districts	6	6	8	20
Number of facilities	306	357	355	1006
Number of children^b^	88,492	53,068	110,255	251,815
Date range of EIR records (including retrospectively entered data)	January 2015-September 2018	January 2015-September 2018	January 2015-September 2018	NA
Date range of EIR introduction	June 2016-March 2017	December 2017-February 2018	July 2017-August 2017	NA
Primary power source, n(col%)^c^				
Grid	102 (37%)	226 (79%)	–	328 (37%)
Solar	87 (31%)	22 (8%)	–	109 (12%)
None	11 (4%)	–	–	11 (1%)
Ownership type, n (col %)^d^				
Private – Faith-based organization	90 (32%)	70 (25%)	37 (11%)	197 (22%)
Public - Government	181 (65%)	201 (71%)	279 (86%)	661 (75%)
Facility type, n (col %)^e^				
Dispensary/health post	218 (78%)	226 (79%)	280 (87%)	724 (82%)
Health center	47 (17%)	45 (16%)	36 (11%)	128 (14%)
Hospital	13 (5%)	14 (5%)	8 (2%)	34 (4%)

^a^Amongst those facilities that input at least one record into the EIR

^b^Children under 2 years as of the time the data were pulled (September 2018)

^c^Facilities missing data on ownership type: in Arusha (*n* = 7), Kilimanjaro (*n* = 14), and Tanga (*n* = 8)

^d^Facilities missing data on primary power source: in Arusha (*n* = 78), Kilimanjaro (*n* = 37), and Tanga (*n* = 327)

^e^Facilities missing data on facility type: in Arusha (*n* = 0), Kilimanjaro (*n* = 14), and Tanga (*n* = 36)

### Individual-level vaccination summaries

**Fig. 3:**
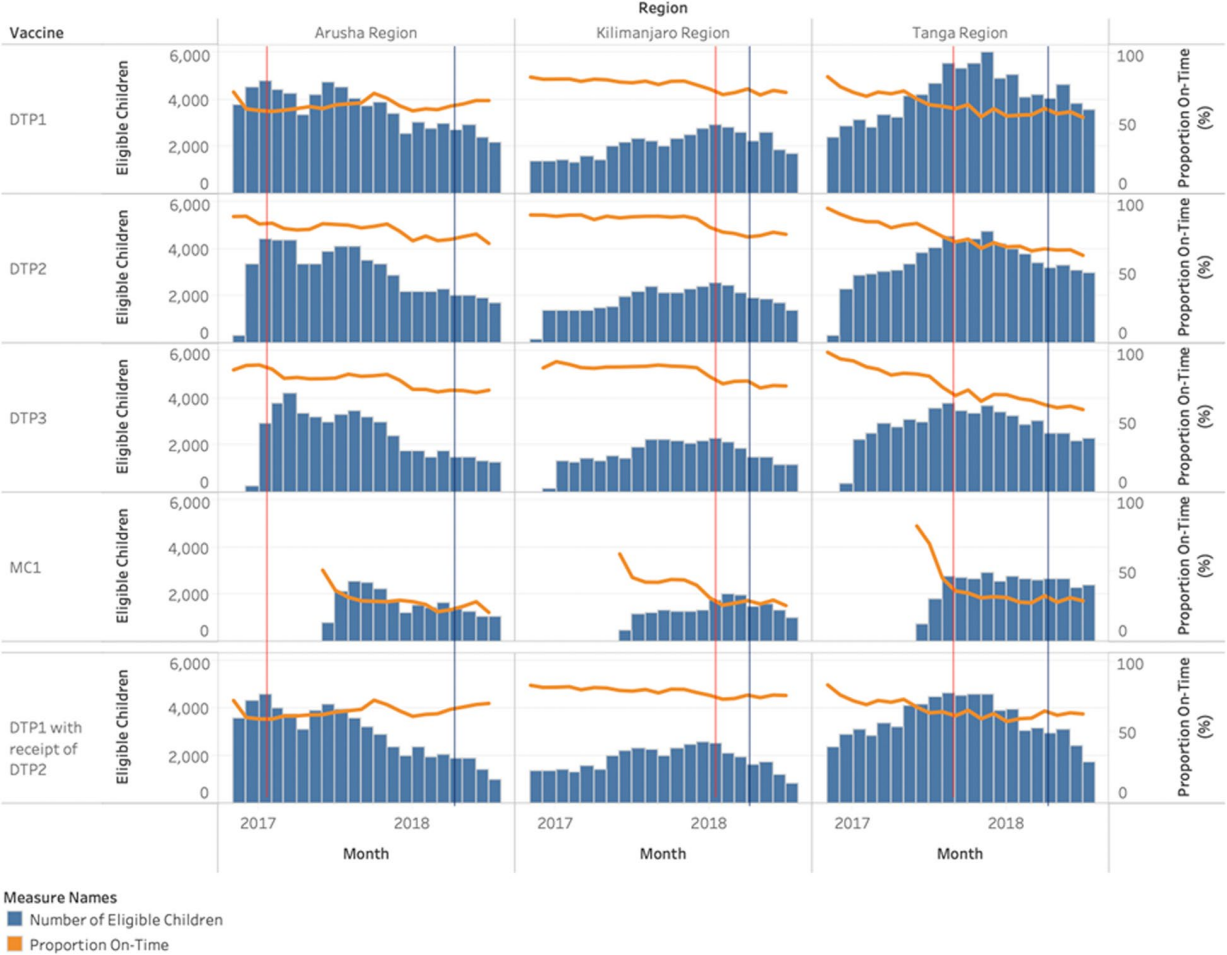
Number of children with a documented vaccination and proportion vaccinated on-time by vaccine type and region, Tanzania, November 2016-July 2018* *Red line indicates when the EIR was introduced (approximately); blue line indicates when the SMS reminders were introduced

**Fig. 4:**
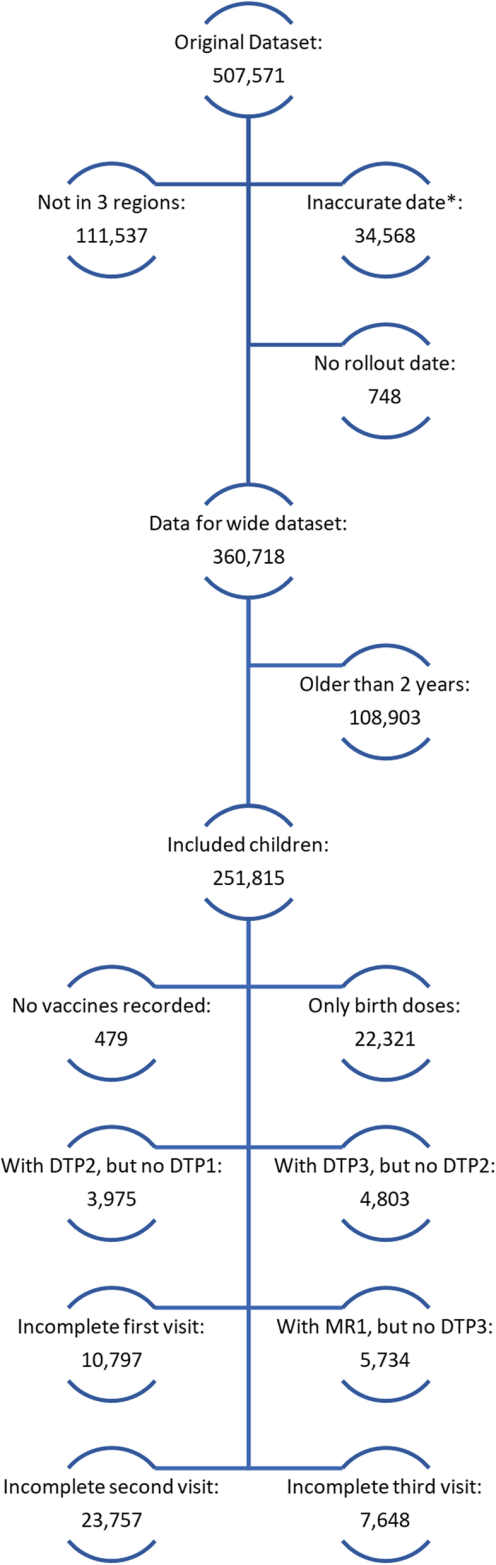
Incomplete or missing vaccination records among all children registered in the EIR* *For the selected vaccines of interest and includes doses administered after data pull and doses administered before a date-of-birth, which were removed for the analysis

There were 246,940 children eligible for DPT1, 243,871 for DTP2, 236,691 for DTP3, and 170,279 for MCV1 vaccines (Table 2). The DTP1 vaccine was most frequently on-time (57.9%) with a mean of 13 days after the scheduled date, while MCV1 vaccine was least likely to be on-time (15.8%), with a mean of 25 days off-schedule. For all vaccine types, vaccinations were more frequently on-time or early before introduction of the EIR. Differences between pre- and post-EIR time periods were observed for all vaccines, with DTP3 and MCV1 vaccines having the largest differences. For all vaccine types, on average more vaccinations were recorded post-EIR introduction per month, with an average of 11 doses administered per month per facility pre-EIR compared to 16 doses post-EIR for DTP1. Fewer doses of MCV1 were administered pre-EIR, 6 versus 15 post-EIR. There were no differences in the direction of the trends observed across regions. Variations by vaccine were observed, with the proportion of vaccinations on-time generally decreasing over time, but a slightly positive trend was observed in Arusha for DTP1 (Fig. 3). Amongst all children under 2 years at the time of the data pull, 479 (0.2%) had no recorded vaccinations, 22,321 (8.9%) received no doses after their birth doses, 8778 (3.5%) had DTP2 or DTP3 recorded, but not the previous dose (these were dropped from the analysis), and 5734 (2.3%) had a recorded MCV1 vaccine, but no DTP3 recorded. There were 10,797 (4.3%) children receiving at least one, but not all of the vaccines recommended at their 6-week visit, 23,757 (9.4%) at the 10-week visit, and 7648 (3.0%) at the 14-week visit. (Fig. 4).

**Table 2:** On-time vaccinations, days off-schedule, and weekly vaccinations per facility, by vaccine type and region

Vaccine	Characteristics	Arusha	Kilimanjaro	Tanga	All Regions
**DTP1**	Number of eligible children^a^	87,084	51,729	108,127	246,940
	Vaccinated (%)	77,988 (89.6)	45,854 (88.6)	92,720 (85.8)	216,562 (87.7)
	On-time vaccination (%)	49,349 (56.7)	35,384 (68.4)	58,210 (53.8)	142,943 (57.9)
	Mean number of days off-schedule, (SD)	13 (34)	9 (32)	16 (47)	13 (40)
	Early vaccination, pre-EIR (%)	719 (5.5)	1149 (4.1)	1756 (4.4)	3624 (4.4)
	Early vaccination, post-EIR (%)	2200 (3.0)	686 (2.9)	2900 (4.3)	5786 (3.5)
	On-time vaccination, pre-EIR (%)	7881 (60.5)	22,439 (79.1)	25,643 (63.9)	55,963 (68.7)
	On-time vaccination, post-EIR (%)	41,468 (56.0)	12,945 (55.4)	32,567(47.9)	86,980 (52.6)
	Mean vaccinations administered per facility per month- pre-EIR (SD)	17 (29)	7 (8)	12 (15)	11 (15)
	Mean vaccinations administered per facility per month- post-EIR (SD)	16 (26)	11 (18)	18 (22)	16 (24)
**DTP2**	Number of eligible children^a^	86,836	50,687	106,348	243,871
	Vaccinated (%)	63,132 (72.7)	39,394 (77.7)	74,203 (69.8)	176,729 (72.5)
	On-time vaccination (%)	51,565 (59.4)	33,764 (66.6)	55,549 (52.2)	140,878 (57.8)
	Mean number of days off-schedule, (SD)	7 (25)	5 (19)	9 (29)	8 (25)
	Early vaccination, pre-EIR (%)	445 (4.4)	801 (3.1)	1241 (3.6)	2487 (3.5)
	Early vaccination, post-EIR (%)	2101 (2.7)	655 (2.6)	2496 (3.5)	5253 (3.0)
	On-time vaccination, pre-EIR (%)	7834 (77.5)	22,423 (87.1)	25,236 (72.7)	55,493 (78.6)
	On-time vaccination, post-EIR (%)	43,731 (57.0)	11,341 (45.5)	30,313 (42.3)	85,385 (51.4)
	Mean vaccinations administered per facility per month- pre-EIR (SD)	17 (30)	7 (8)	12 (15)	10 (15)
	Mean vaccinations administered per facility per month- post-EIR (SD)	17 (27)	11 (18)	19 (23)	16 (24)
**DTP3**	Number of eligible children^a^	84,923	48,860	102,908	236,691
	Vaccinated (%)	50,273 (59.2)	34,160 (70.0)	60,174 (58.5)	144,607 (61.1)
	On-time vaccination (%)	40,713 (47.9)	29,200 (59.8)	44,185 (42.9)	114,098 (48.2)
	Mean number of days off-schedule, (SD)	9 (32)	6 (25)	13 (38)	10 (33)
	Early vaccination, pre-EIR (%)	288 (4.0)	775 (3.4)	966 (3.4)	2029 (3.4)
	Early vaccination, post-EIR (%)	1742 (2.2)	566 (2.2)	2071 (2.8)	4379 (2.5)
	On-time vaccination, pre-EIR (%)	5148 (71.0)	19,758 (86.0)	20,375 (71.0)	45,281 (76.8)
	On-time vaccination, post-EIR (%)	35,565 (45.8)	9442 (36.5)	23,810 (32.1)	68,817 (38.7)
	Mean vaccinations administered per facility per month- pre-EIR (SD)	16 (29)	7 (8)	11 (14)	9 (13)
	Mean vaccinations administered per facility per month- post-EIR (SD)	17 (27)	11 (18)	19 (23)	17 (24)
**MCV1**	Number of eligible children^a^	65,848	30,978	73,453	170,279
	Vaccinated (%)	23,876 (36.3)	20,343 (65.7)	37,850 (51.5)	82,069 (48.2)
	On-time vaccination (%)	6832 (10.4)	6953 (22.4)	13,194 (18.0)	26,979 (15.8)
	Mean number of days off-schedule, (SD)	27 (49)	21 (41)	26 (47)	25 (47)
	Early vaccination, pre-EIR (%)	1 (100)	1191 (12.8)	507 (8.5)	1699 (11.1)
	Early vaccination, post-EIR (%)	2721 (4.1)	1166 (5.4)	2810 (4.2)	6697 (4.3)
	On-time vaccination, pre- EIR (%)	0 (0)	3746 (40.2)	3084 (51.8)	6833 (44.7)
	On-time vaccination, post- EIR (%)	6832 (10.4)	3206 (14.8)	10,108 (15.0)	20,146 (13.0)
	Mean vaccinations administered per facility per month- pre-EIR (SD)	1 (NA)	5 (6)	7 (10)	6 (7)
	Mean vaccinations administered per facility per month- post-EIR (SD)	17 (28)	9 (12)	17 (21)	15 (23)

^a^Children over the recommended age of vaccination (6 weeks for DTP1, 10 weeks for DTP2, 14 weeks for DTP3, and 9 months for MCV1) and under 2 years as of the time the data were pulled (September 2018)

### Model findings

**Fig. 5:**
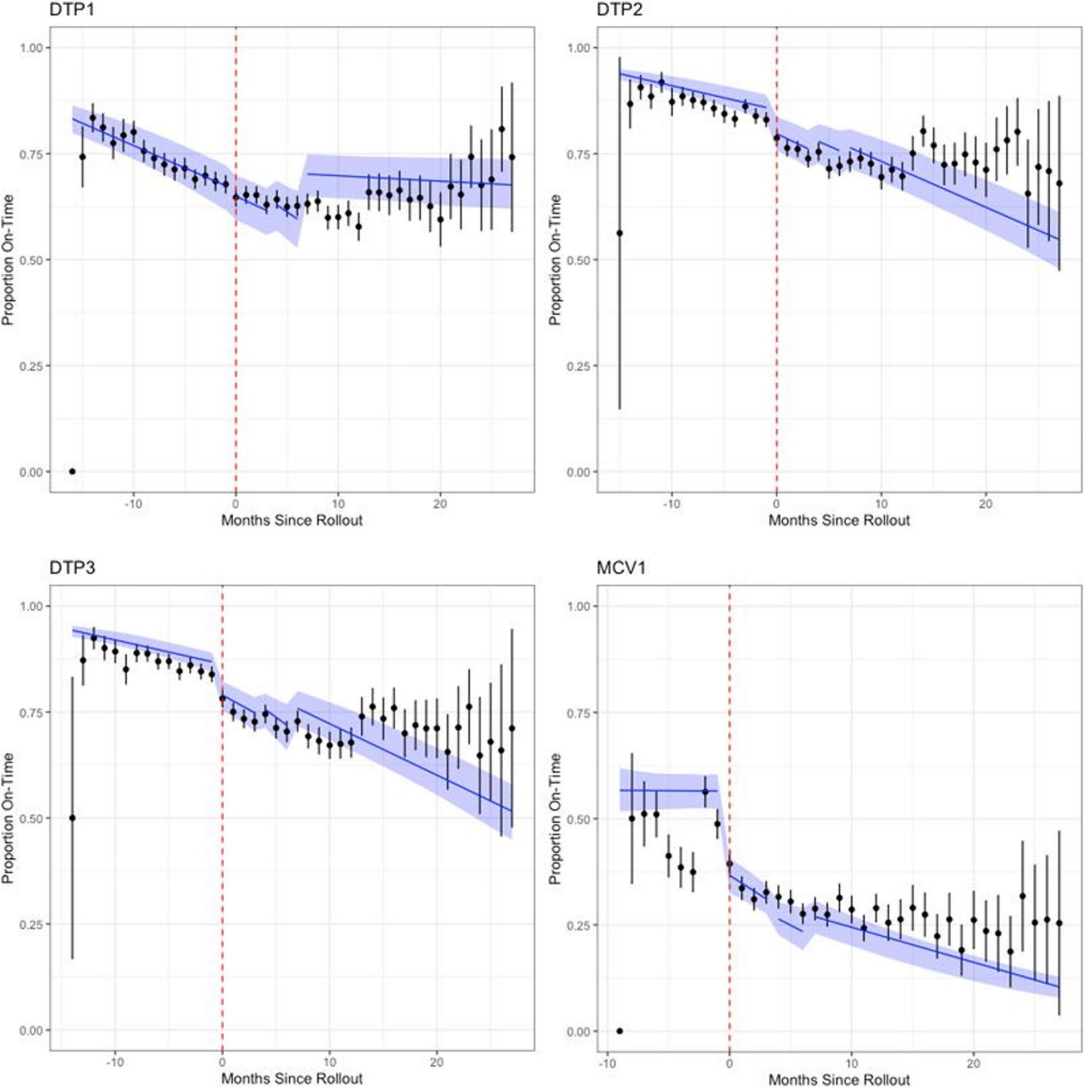
Regression results for primary model* *Red dotted line indicates EIR-introduction date; black dots indicate the average proportion of vaccinations per facility per month with the black lines indicating their associated 95% confidence intervals; blue lines indicate the change in slope for the proportion of children vaccinated on-time per month per facility

The binomial regression models estimated significant differences in proportion of on-time vaccinations before and after rollout of the EIR, based on level changes (Table 3 and Fig. 5). Compared to the pre-EIR time period, the likelihood of an on-time DTP1 vaccination decreased by 5% (OR:0.95, 95% CI: 0.90-0.99) in the first 3 months following EIR-introduction, but increased 14% (OR:1.15 95% CI: 1.07-1.22) and 25% (OR:1.26, 95% CI: 1.15-1.35) 4-6 months and > 6 months post-EIR, respectively. For DTP2, the likelihood of on-time vaccinations was lower post-EIR, compared to pre-EIR, although the reduction decreased over time with 32% (OR:0.68, 95% CI: 0.62-0.75) reduced likelihood 0-3 months post-EIR, 22% (OR:0.78, 95% CI: 0.68-0.88) 4-6 months post-EIR, and 20% (OR:0.80, 95% CI: 0.68-0.93) post-EIR > 6 months after. A similar trend in level changes was observed for DTP3, with a consistent lower likelihood of on-time vaccination post-EIR introduction, but with improvements seen over time. For MCV1 vaccinations, compared to the pre-EIR time period, the likelihood of on-time vaccination was 55% lower (OR:0.45, 95% CI: 0.36-0.53) 0-3 months post-introduction, 62% lower (OR:0.38, 95% CI: 0.23-0.54) 4-6 months, and 57% lower (OR:0.43, 95% CI: 0.25-0.61) > 6 months post-introduction.

**Table 3:** Parameter estimates for likelihood of change in the proportion of on-time vaccinations pre- and post-introduction of an EIR, Tanzania*

	*Dependent variable:*			
	On-Time Vaccination			
	DTP1	DTP2	DTP3	MCV1
	(1)	(2)	(3)	(4)
Baseline monthly change in slope	0.94* (0.94, 0.95)	0.94* (0.93, 0.94)	0.93* (0.92, 0.94)	1.00 (0.98, 1.02)
Level change 0-3 Months after EIR	0.95* (0.90, 0.99)	0.68* (0.62, 0.75)	0.61* (0.54, 0.67)	0.45* (0.36, 0.53)
Level change 4-6 Months after EIR	1.14* (1.07, 1.22)	0.78* (0.68, 0.88)	0.74* (0.62, 0.85)	0.38* (0.23, 0.54)
Level change > 6 Months after EIR	1.25* (1.15, 1.35)	0.80* (0.68, 0.93)	0.75* (0.61, 0.89)	0.43* (0.25, 0.61)
Change in slope 0-3 Months after EIR	1.01 (0.99, 1.03)	1.00 (0.98, 1.02)	0.99 (0.96, 1.02)	0.92* (0.89, 0.96)
Change in slope 4-6 Months after EIR	0.99 (0.96, 1.02)	1.01 (0.97, 1.04)	0.97 (0.93, 1.01)	0.92* (0.88, 0.97)
Change in slope > 6 Months after EIR	1.05* (1.05, 1.06)	1.02* (1.01, 1.03)	1.02* (1.00, 1.03)	0.94* (0.92, 0.97)
Observations	17,092	15,854	14,546	9562

*p < 0.05; Intercept estimates are not presented

Statistically significant changes in the slope of the trend were estimated for > 6 months post-EIR introduction, compared to pre-EIR, for all vaccine types. For DTP1 vaccinations, the likelihood of on-time vaccinations increased by 5% per month (OR:1.05, 95% CI: 1.05-1.06), for DTP2 the likelihood increased by 2% (OR:1.02, 95% CI: 1.01-1.03), for DTP3 the likelihood increased by 2% (OR:1.01, 95% CI: 1.00-1.03), and for MCV1 the likelihood decreased by 6% per month (OR:0.94, 95% CI: 0.92-0.97).

### Findings of sensitivity analysis

**Fig. 6:**
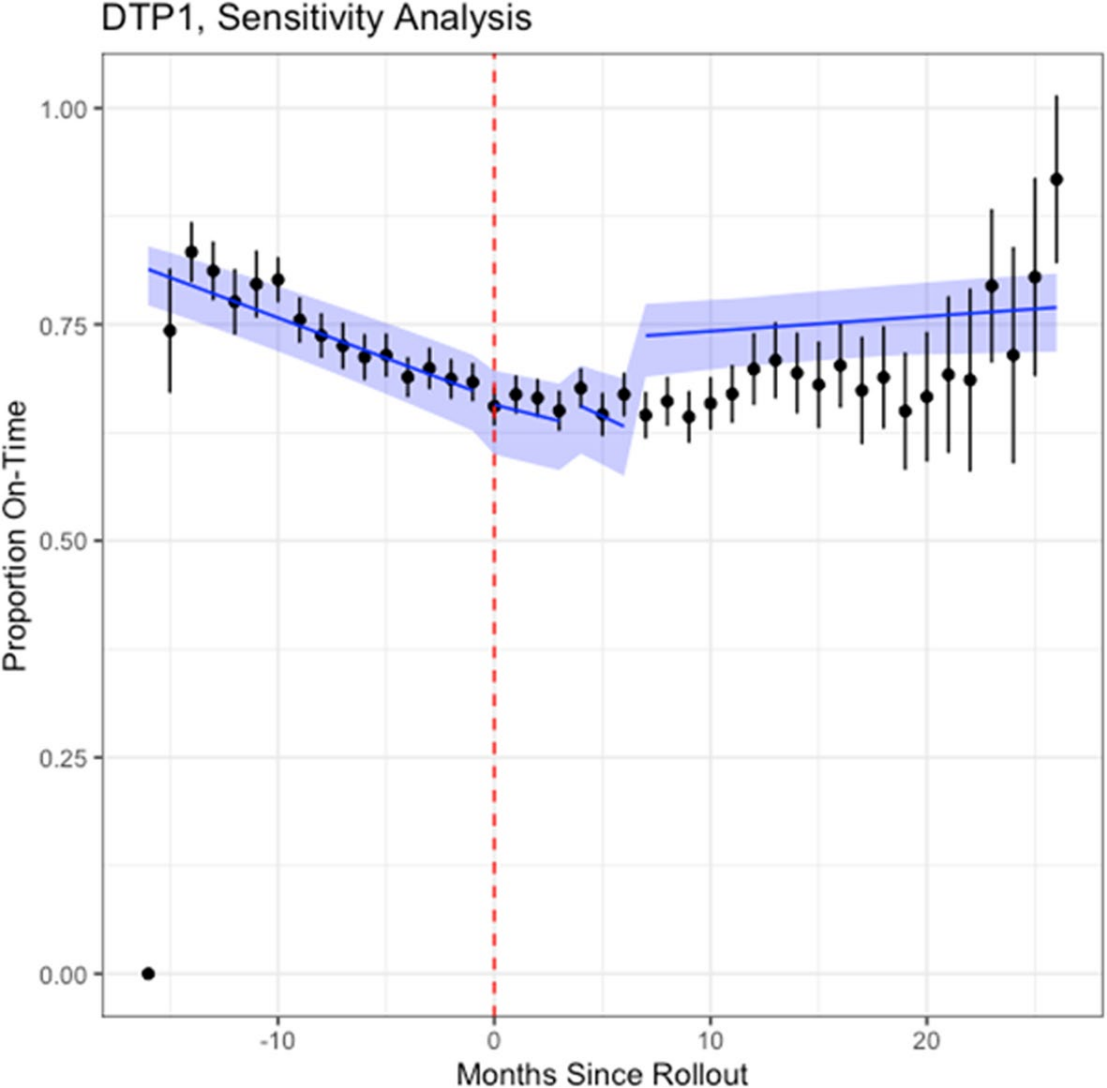
Regression results for sensitivity analysis* *Red dotted line indicates EIR-introduction date; black dots indicate the average proportion of vaccinations per facility per month with the black lines indicating their associated 95% confidence intervals; blue lines indicate the change in slope for the proportion of children vaccinated on-time per month per facility

Changes over time were observed showing a decrease and then roughly a plateau in timeliness following the introduction of the EIR (Fig. 3). We estimated significant differences in the likelihood of on-time DTP1 vaccination post-EIR introduction compared to pre-EIR, with a 19% increased likelihood of being on-time 4-6 months post-EIR (OR: 1.19, 95% CI: 1.11-1.27) and 34% increase > 6 months post-EIR (OR: 1.34, 95% CI: 1.23-1.45) (Table 4 and Fig. 6). Significant increases in the likelihood of on-time vaccinations were estimated for 0-3 months and > 6 months following EIR-introduction, with 2% increase per month (OR: 1.02, 95% CI: 1.00-1.04) and 6% increase per month (OR: 1.06, 95% CI: 1.05-1.07), respectively.

**Table 4:** Parameter estimates for likelihood of change in proportion of on-time DTP1 vaccination pre- and post-introduction of an EIR among children with a documented dose of DTP2 (sensitivity analysis)*

	*Dependent variable:*
	On-Time Vaccination
	DTP1
Baseline monthly change in slope	0.95* (0.95, 0.96)
Level change 0-3 Months after EIR	0.98 (0.93, 1.03)
Level change 4-6 Months after EIR	1.19* (1.11, 1.27)
Level change > 6 Months after EIR	1.34* (1.23, 1.45)
Change in slope 0-3 Months EIR	1.02* (1.00, 1.04)
Change in slope 4-6 Months after EIR	1.00 (0.97, 1.03)
Change in slope > 6 Months after EIR	1.06* (1.05, 1.07)
Observations	15,743

*p < 0.05; Intercept estimates are not presented

## Discussion

### Findings

We observed decreases in the proportion of on-time vaccinations following EIR-introduction. However, we emphasize caution interpreting these findings as additional information is needed to understand if the changes observed reflect true estimates of timeliness or if they reflect “noise” due to incompleteness in EIR vaccination records and biases from the data capture process. From our sensitivity analysis, we observed that among those children receiving DTP2, there were improvements in DTP1 timeliness following EIR-introduction, indicating that our belief about incomplete EIR records may very well be valid and the decreases in timeliness observed from the primary analysis are not accurate.

Upon a crude comparison of our estimates to the most recent Demographic and Health Survey (DHS) conducted in Tanzania for 2015-2016, we found further evidence for inconsistent EIR data entry if we assume true immunization coverage did not vary much between 2015 and 2018. The DHS defines timeliness as children receiving recommended vaccinations before age 12 months [29]. We compared the survey estimates to EIR estimates using the same timeliness definition, and observed that prior to EIR-introduction national timeliness estimates were comparable across data sources, for instance DTP1 timeliness was 96.5% in the EIR and estimated to be 96.6% nationally from DHS (See Additional file 1). However, we found that estimates for DTP2, DTP3, and MCV1 decreased following EIR-introduction, suggesting inconsistent data entry post-EIR. We should note that DHSs use information recorded from vaccination cards and parental recall, these data have been found to be unreliable when compared to medical records, and therefore the DHS should not be considered the gold standard for our comparison [30].

Our research group previously noted, “completeness and quality of data input into a system dictates the accuracy of the estimates generated by the system, contingent on the system’s design, user compliance, and system maintenance”, and that “calculating accurate estimates of performance measures using EIR data will likely remain elusive until the challenges” have been addressed [31]. Implementation challenges that may have affected the completeness and/or accuracy of the data in our study included: inconsistent use of the EIR over time, the official requirement of completing dual data entry with the paper record remaining the official record potentially causing HCWs to ensure paper records were more complete than EIR records, inconsistent use of unique patient identifiers causing individuals to have multiple IDs, or poor data entry practices leading to inaccuracies due to workflow or training issues [32, 33]. Inconsistent use of the system is further complicated by facilities using different methods for entering data retrospectively and documenting outreach sessions, as well as staffing changes. During early to mid-2017, the Tanzanian government began restricting public employees who could not prove they had completed their secondary education via a paper certificate. This resulted in the loss of approximately 10,000 employees, including HCWs, who were fired from their positions if they could not present the certificate [34]. The drop in the workforce likely affected the capacity for HCWs to consistently use the EIR during our study period. Additionally, there were potentially server-side issues that could have prevented all data from being made available due to the server timing out or being overloaded. Also, the EIR’s validation rules may not have been functioning correctly, since we found many records with implausible vaccination dates based on the date of birth. Studies based in other countries have found similar challenges, particularly a high rate of under-reporting which causes underestimation of vaccination coverage, low IT literacy needed for adoption of DHI, and poor integration of the DHI within the existing health system [7, 35–37].

Considering our study team found no alternative reason for true vaccination timeliness to decrease following EIR-introduction after consulting with implementers and MOH staff, and that our results did not align with survey data, the most likely explanation is that our primary analysis results suffer from presumed threats to validity. Rather than timeliness decreasing at a population-level, it seems more likely that the results reflect the EIR implementation challenges described above. However, some facilities may have captured more accurate information in the EIR than previously captured by paper-based tools, and post-EIR, we may have observed true vaccination timeliness previously uncaptured in surveys and other assessment methods, which is useful when reviewing the descriptive results, but is less useful for our time-series analysis. It will be important to reanalyze the data again in a couple of years to understand if the trends have changed due to improvements in EIR-use. Additionally, these threats to validity also further violate the exchangeability assumption needed to assess impact using an interrupted time-series analysis, where we cannot assume that children’s records entered retrospectively versus prospectively are comparable. There may be multiple potential explanations for the trends seen in our data that would require primary data collection to confirm.

### Can digital health interventions improve health outcomes?

We have outlined the potential implementation challenges that may have impacted our analysis; however, it is worth revisiting our theoretical framework to understand where gaps in adoption and use of the EIR may have occurred and subsequently affected vaccination timeliness. EIRs are implemented within complex health systems and require HCW activities to accommodate new workflows that incorporate the tool so their effectiveness relies on how well they are designed, developed, implemented, and used [21]. Consistent entry of data into an EIR may be dependent on HCW competency in using DHI tools, a facility’s internet and electricity connectivity, dual data entry, and HCW motivation, all of which could impact the completeness of EIR records.

Simply having HCWs utilize an electronic tool will not on its own increase timeliness of vaccinations; our causal linkage diagram shows that HCWs would have to use the information in the EIR to encourage caregivers to bring children on-time for their next scheduled immunization appointment and follow-up with defaulters. A realist review found similar findings, with only moderate to low-certainty evidence of EIRs improving data use amongst district- and facility-level staff [21, 38]. Although in Tanzania HCWs were trained to follow-up using EIR information, it is unclear how consistent this was performed. We did not estimate the effect of the SMS-reminders component of the intervention due to the limited follow-up time, but this is a future area of research as it could have affected timeliness. Finally, the length of time to observe behavior change is unknown as it likely varies by caregiver, HCW, and facility. Our assumptions may have underestimated the amount of time needed for HCWs’ behaviors to change.

Furthermore, these challenges beg the question: if these systems make no impact on timeliness, should we invest in them? Improvements have been seen in other settings, an EIR and SMS-reminder system was successfully deployed in Vietnam, where improvement in vaccination timeliness was observed 2 years following system introduction [6]. Additionally, in high-income settings, improvements in coverage and timeliness have been observed due to electronic systems [5]. However, it is worth first asking whether health-related outcomes are the best measures to quantify the impact of these digital systems. Considering the large footprint required for deployment, that often involves cross-team collaborations and implicates staff at each health system level, these systems have additional effects and impacts that are not captured by patient health outcome metrics but may still improve healthcare provision. Digital systems can provide a secure location for record storage, increase patient trust in the healthcare system, improve data quality and accessibility, and can reduce the burden of data management activities, freeing up time for staff to focus on patient care [39]. As the health benefits of DHI may take 3-13 years to be observed, the importance of these more proximal process outcomes should be acknowledged and these metrics used in DHI evaluations [40].

### Study strengths

Our study took advantage of the opportunity to use individual-level RHIS data to conduct a quasi-experimental analysis. We were able to showcase the utility and power of these data for answering an implementation science research question by developing appropriate performance metrics within the Tanzanian context that considered changes over time, clinician practices at the facility and district levels, and the cohort of children we expected to see most affected by the EIR’s introduction. The study design process required that the research and implementation teams worked closely together to create a model that would accurately capture EIR introduction and use among facilities and provide interpretable findings.

### Limitations

We encountered numerous challenges using these data to answer our research question, mostly due to EIR-implementation complexities. Upon review of the challenges, we considered some of them to be natural to the process of implementing a new DHI. However, using these data “as-is” for our analysis was difficult due to poor data quality, necessitating a need for a clear understanding of the implementation setting and challenges to continuous data entry and use. There are several important contextual factors which were not accounted for in our models. Because time was centered on EIR introduction date, we were unable to account for the timing of other events or secular trends, such as the public employee dismissal, that could have potentially impacted vaccination timeliness, including changes made to the intervention package. Additionally, without further verification through other data sources and observations, it is difficult to know the level of completeness of the EIR data or when to consider the data to have “normalized”. We also recognized that our study could be affected by system impacts not accounted for in the analysis and other unmeasured confounders. A key limitation of this study is that we were unable to assess immunization coverage as an outcome since we lacked data on the full denominator population of children eligible for vaccination in the community. Future analyses should assess vaccination drop-out, comparing the number of children receiving the first to the third dose, as this is a better measure of timeliness because it accounts for the entire vaccine series and allows changes in timeliness to be measured at the individual-level, rather than facility-level.

## Conclusions

We assessed changes in vaccination timeliness following EIR-introduction and found that timeliness decreased over time, likely due to inconsistent data entry and use of the EIR, rather than a true decrease in the population. To interpret our findings more accurately, contextual information about EIR implementation would have helped to provide a comprehensive understanding of the validity of this finding. EIRs have the potential to improve vaccination timeliness but need to be used consistently to provide accurate metrics on target populations. Use of individual-level RHIS data generated from these systems can provide greater insight into immunization program performance and ultimately help reduce gaps in vaccination coverage once implementation challenges are overcome.

## Supplementary Information

**Additional file 1: Supplementary Table 1.** Comparison of immunization coverage between data sources. **Supplementary Table 2.** Tanzania immunization schedule primary and adolescent infant vaccination schedule.

## Data Availability

The datasets generated and/or analysed during the current study are not publicly available because they are owned by the Tanzania Ministry of Health, but are available from the corresponding author on reasonable request.

## Abbreviations

BID: Better Immunization Data
DHI: Digital health intervention
DHS: Demographic and Health Survey
DTP: Diphtheria, tetanus, and whole-cell pertussis, hepatitis B, and *Haemophilus influenzae* type b vaccine
EIR: Electronic immunization registry
HCW: Healthcare worker
LMIC: Low- and middle-income countries
MC: Measles containing vaccine
MOH: Ministry of health
POC: Point-of-care
RHIS: Routine health information system
SMS: Short-message service
TImR: Tanzania Immunization Registry
UAG: User-Advisory Group
