# Supplementary material for: Changes in on-time vaccination following the introduction of an electronic immunization registry, Tanzania 2016-2018: interrupted time-series analysis
Source: BMC Health Serv Res. 2022 Sep 20;22:1175. doi: 10.1186/s12913-022-08504-2 (PMC9485799; doi:10.1186/s12913-022-08504-2)
Supplement: Supplementary file 1 — Additional file 1: Supplementary Table 1. Comparison of immunization coverage between data sources. Supplementary Table 2. Tanzania immunization schedule primary and adolescent infant vaccination schedule. [file 12913_2022_8504_MOESM1_ESM.docx]

**Supplementary Table 1. Comparison of Immunization Coverage between data sources**

|  | | **Pre-introduction of the EIR** | **Post-introduction of the EIR** | **Overall** |  | **Demographic and Health Survey and Malaria Indicator Survey 2015-16** |
| --- | --- | --- | --- | --- | --- | --- |
| **Amongst children receiving vax before age 12 months** | | | | | | |
| **All Regions** | **DTP1** | 96.5 | 93.0 | 95.0 |  | 96.6 |
|  | **DTP2** | 92.6 | 81.5 | 87.1 |  | 93.4 |
|  | **DTP3** | 89.1 | 68.4 | 77.5 |  | 87.7 |
|  | **MR1** | 90.1 | 47.1 | 52.2 |  | 78.0 |

**Supplementary Table 2. Tanzania Immunization Schedule**

**Primary and Adolescent Infant Vaccination Schedule**

| **Vaccine** | **Description** | **Schedule** |
| --- | --- | --- |
| BCG | Bacille Calmette-Guerin vaccine | Birth |
| OPV | Oral polio vaccine | Birth; 6, 10, 14 weeks |
| Rotavirus | Rotavirus vaccine | 6, 10 weeks |
| DTwPHibHepB | Diphtheria and Tetanus and Pertussis and Haemophilus influenzae and Hepatitis B vaccine | 6, 10, 14 weeks |
| Pneumo_conj | Pneumococcal conjugate vaccine | 6, 10, 14 weeks |
| MR | Measles and rubella vaccine | 9, 18 months |
